# Exploring the human experience of congenital aniridia: A narrative medicine approach

**DOI:** 10.1177/11206721251407851

**Published:** 2025-12-16

**Authors:** Chiara Ancona, Maria Giulia Marini, Francesco Minetti, Mária Csidey, Erika Maka, Tanja Stachon, Fabian Norbert Fries, Barbara Poli, Ivana Kildsgaard, Dominique Bremond-Gignac, Nóra Szentmáry, Neil Lagali, Vito Romano

**Affiliations:** 1 Eye Unit, Department of Medical and Surgical Specialties, Radiological Sciences, and Public Health, University of Brescia, Brescia, Italy; 2 18606 Ospedale Fatebenefratelli Oftalmico, Milano, Italy; 3 Health Care, 72270 ISTUD Foundation, Milan, Italy; 4 Department of Ophthalmology, 37637 Semmelweis University, Budapest, Hungary; 5 Department of Opthalmology, 37643 Heim Pál National Pediatric Institute, Budapest, Hungary; 6 Dr. Rolf M. Schwiete Center for Limbal Stem Cell and Aniridia Research, 9379 Saarland University, Homburg/Saar, Germany; 7 Department of Ophthalmology, Saarland University Medical Center, Homburg/Saar, Germany; 8 Aniridia Europe, Sandefjord, Norway; 9 Aniridia Sweden, Stockholm, Sweden; 10 Ophthalmology Department, Necker Enfants Malades University Hospital, AP-HP, 246596 Paris Cité University, Paris, France; 11 INSERM UMRS1138, Team 17, From Physiopathology of Ocular Diseases to Clinical Development, Sorbonne Paris Cité University, Centre de Recherche des Cordeliers, Paris, France; 12 Department of Biomedical and Clinical Sciences, Faculty of Medicine, 4566 Linköping University, Linköping, Sweden; 13 Department of Ophthalmology, 375794 Sørlandet Hospital Arendal, Arendal, Norway

**Keywords:** Congenital aniridia, narrative medicine, narratives, patients, caregivers

## Abstract

**Purpose:**

To explore the emotional and social burden of congenital aniridia in patients and caregivers through narrative medicine.

**Methods:**

Patients with congenital aniridia and their caregivers were enrolled in a multicentric observational study. Sociodemographic data and personal narratives were collected through interviews or written accounts. A qualitative analysis was conducted and overarching themes identified.

**Results:**

A total of 57 narratives were collected (31 patients, 27 caregivers). Participants reported visiting an average of 5.1 ± 5.9 healthcare facilities over their lifetime. Aniridia impacted work or school life in 39% of patients and 58% of caregivers. Most narratives focused on the lived experience of illness (95% patients, 81% caregivers) or on social perception (48% and 46%), rather than on clinical aspects (10% and 23%). Adaptation to the disease was mainly stable or progressive among adults. Emerging themes included independence, feeling misunderstood, and bullying. Caregivers emphasized the lack of specialized centers, standardized protocols, adequately trained professionals, psychological and practical support, and the lack of humanity among doctors.

**Conclusions:**

Congenital aniridia leads to progressive visual impairment with significant emotional and social consequences. Narrative medicine offers valuable insight into patients’ and caregivers’ lived experiences, highlighting unmet needs and concerns often overlooked in conventional clinical practice.

## Introduction

Congenital aniridia is a rare genetic disorder with a prevalence of 1:72,000.^1^ Two-thirds of cases are autosomal dominant due to PAX6 gene mutations, while one-third are sporadic, linked to de novo deletions in chromosome 11p13.^2^ The condition is a panocular disorder as it involves more than iris malformation; it often includes foveal hypoplasia, cataract, corneal opacity, and glaucoma. The involvement of the WT1 gene adjacent to PAX6 can result in the development of WAGR syndrome, which is characterized by the presence of Wilms tumor, aniridia, genitourinary anomalies and retardation. Another rare variant of aniridia with an unclear inheritance is known as Gillespie syndrome. This is characterized by aniridia, cerebellar ataxia, ptosis and intellectual disability.^2^ Even when overt syndromes like WAGR or Gillespie are ruled out, aniridia often extends beyond the eyes, affecting other organs. For this reason it is increasingly referred to as aniridia syndrome.^2^

Iris hypoplasia ranges from subtle to severe. Visual acuity is consistently reduced, primarily due to foveal hypoplasia, with dry eye disease, limbal stem cell deficiency, and glaucoma also contributing.^1,3^ Symptoms and signs begin in infancy with nystagmus and “black eyes,” (iris hypoplasia) with the causative mutation confirmed via genetic testing. Treatments like cataract and glaucoma surgeries carry risks due to impaired wound healing and limbal stem cell insufficiency of the cornea.^4^

Patients require regular eye exams for refractive corrections, corneal monitoring, and glaucoma screening. Surveillance for Wilms tumor is necessary in syndromic cases.^5^ Management includes tinted lenses or photochromic lenses to reduce light sensitivity, artificial tears, and intraocular pressure-lowering drops. As visual acuity declines, the provision of assistive devices becomes crucial for the wellbeing of these individuals.^6^

The majority of aniridia research is concerned with identifying the causes of the disease, its underlying pathophysiology, the symptoms that manifest, the diagnostic procedures that can be employed, and the potential treatments that could be offered. However, current research offers little insight into the lived experiences of individuals with aniridia or the associated emotional and social challenges they and their caregivers face. This gap is particularly problematic given the limitations of traditional clinical measures to fully capture the condition's personal and social impacts.

Narrative medicine (NM), a collection of the narratives of patients, caregivers and healthcare professionals, represents a response to the need of approach the person holistically, enabling a deeper understanding of how the experience of the disease affects the individual's everyday life, their relationships with others, and their broader social experience.^7^ It is important to clarify that narrative medicine is distinct from a narrative review or from generic qualitative methods. Whereas a narrative review summarizes published literature, and qualitative research may rely on interviews or focus groups without a specific theoretical lens, narrative medicine is grounded in the medical humanities. It systematically collects and interprets personal stories to capture the illness experience, complementing biomedical knowledge with lived perspectives. Previous applications of NM in rare diseases and ophthalmology have shown its value in illuminating psychosocial dimensions of illness, supporting patient-centered care and guiding clinical practice.^8–10^

NM addresses this need by integrating disease-centered perspectives with illness- and sickness-centered viewpoints, exploring how conditions affect individuals’ emotional, social, and relational networks. NM provides critical insights into the hidden dimensions of illness that conventional metrics cannot quantify, such as independence, social stigma, and bullying—macro-themes that are central to the aniridic experience.^11–13^

The project IRIS – Aniridia Stories: investigating congenital aniridia through narrative medicine, aims to understand the illness burden related to this condition through narratives of patients with congenital aniridia and their caregivers. The project intended to (1) explore the daily experiences of individuals with congenital aniridia within educational, occupational, and social settings, identifying both coping strategies and obstacles related to the illness burden; (2) uncover new insights into quality of life, care pathways, and other factors influencing the daily lives of patients and their caregivers; (3) delineate the burden of illness by assessing its direct and indirect social impacts, and (4) encourage personal, social, and institutional resources to enhance the daily lives of patients with congenital aniridia and their caregivers.

## Methods

### Study design and approvals

The project was conducted as part of a European networking project (COST Action CA-18116 ANIRIDIA NET), where aniridia patient associations across Europe were invited to participate by identifying and contacting members (those with aniridia and/or their caregivers). Those who gave informed consent were included. This study followed the tenets of the Declaration of Helsinki and was conducted after approvals were obtained from the ethical committee of Spedali Civili in Brescia, Italy -study NP 5830 and the amendment 0005985/24 approved from the ethical committee IRCCS Fondazione Policlinico San Matteo in Pavia, Italy. Subjects participated and completed the narratives between August and November 2023.

In June 2023 the steering committee composed of seven members including ophthalmologists, researchers, and representatives of the Aniridia Europe association participated in an online meeting with researchers from Istituto Studi Direzionali (ISTUD), Healthcare Area, to discuss the project's goals and design. They elaborated and refined illness plots and questionnaires addressed to patients, according to their age, and to caregivers.

The illness plots represented the tale of illness as a result of the completion of narrative prompts. They were characterized by narrative stimuli, established on research conducted in the field of linguistics, following the Natural Semantic Metalanguage approach: evocative words that are as neutral and universal as possible. Furthermore, a chronological order was employed to identify the evolution of the experience of being affected by congenital aniridia and undergoing treatment for it.^14,15^ Similarly, validated questionnaires were identified through a systematic literature search to construct the survey on quality of life and illness burden.

The investigation tools (illness plots and questionnaires) were provided in English (see Appendix). Involved researchers were asked to translate the investigation tools into their own languages.

### Data collection

Patients and caregivers were invited to complete narrative prompts in their native language, selected according to the role of the caregiver or patient and the age of the patients, either in written form or through an in-person or video interview conducted by one or two researchers for each country. The interviewer had the option of either recording the dialogue or transcribing it, with the aim of interrupting the respondent as little as possible. There was no fixed time limit for the oral interviews, which typically lasted about an hour. The underlying idea was to give the interviewee the freedom to express themselves without time constraints. The same principle was applied to those who preferred to complete the narratives in written form.

Sociodemographic data were also gathered according to age and role concurrently with the completion of illness plots, by answering the questionnaire on quality of life. The narratives were translated into English by the research team and anonymously collected on the Alchemer platform, after which they were downloaded as Microsoft Excel spreadsheets.

### Analysis

The sociodemographic data were subjected to descriptive statistical analysis. Subsequently, a qualitative analysis was conducted on the narratives.

The analysis involved an iterative coding process. Two researchers (CA, FM), narrative medicine experts, independently coded the narratives after the translation, identifying recurring patterns and themes. Coding discrepancies were discussed in team meetings until consensus was reached. Reliability was ensured through double-coding of a subset of narratives and agreement discussions across the multidisciplinary research team. This process aligns with COREQ recommendations for transparent reporting of qualitative research.

The data were classified in accordance with the categorizations proposed by Kleinman, Launer and Robinson.^11,16^ The former designation refers to aniridia as a disease, illness, or sickness, corresponding to the physical pathology, the individual experience of being affected by aniridia and the social experience of living with the condition, respectively. Two aspects can coexist in any narrative as multiple perspectives may emerge simultaneously. The second analyzes the coping abilities of patients and caregivers. Indeed, in Launer and Robinson's classification the attitude towards aniridia is considered over time, with the definition of the condition being regressive, stable or progressive, depending on whether there is a worsening, unaltered or better approach, respectively. Similarly in this case, two aspects can coexist concurrently, as multiple attitudes may be present, provided that they are not in opposition to one another. Subsequently, macro-themes were identified that transcended the domains of both patients and caregivers, in addition to caregiver-specific macro-themes.

The study relied on convenience sampling, and therefore the cohort may not be fully representative of the broader population living with congenital aniridia. Both descriptive and comparative statistical analyses should thus be interpreted with caution, as they reflect the experiences of those who participated rather than population-level estimates.

## Results

### Sociodemographic aspects

Six countries (Austria, France, Germany, Hungary, Italy, Sweden) participated in the project. Fifty-seven narratives were collected, 31 narratives of patients (five patient narratives from individuals aged between 8 and 11 years old, five from those aged between 12 and 18 years old, and 21 from those aged 18 years old or above) and 26 caregivers. The mean age of patients was 32.5 ± 16.7 (9–56 years) and for caregivers it was 43.6 ± 7.5 years (30–67 years) (Supplementary Tables 1, 2, 3).

28% of respondents had a specialized center for aniridia in the same town where they lived. Respondents visited an average of 5.1 ± 5.9 (range 1–40) different healthcare facilities during their lifetime. Each year an average of 560 ± 932 euros was spent on healthcare visits, with 60% of respondents having additional costs for drugs, transport and aids. For 39% of patients and 58% of caregivers, aniridia had an impact on their work situation or they lost days of work or school because of aniridia.

Together with sociodemographic aspects, adults and caregivers’ narratives were examined and the results presented in two main sections. The first section analyzed aniridia through narrative classifications. The second section identified macro-themes which emerged from the interviews.

Considering the limited number of patients under the age of 18 (with five under the age of 12 and five between the ages of 12 and 17), and the less articulate nature of the prompts administered to these patients, a greater proportion of the analysis was focused on the narratives provided by adult patients and caregivers.

### Classifications

**Figure 1.**
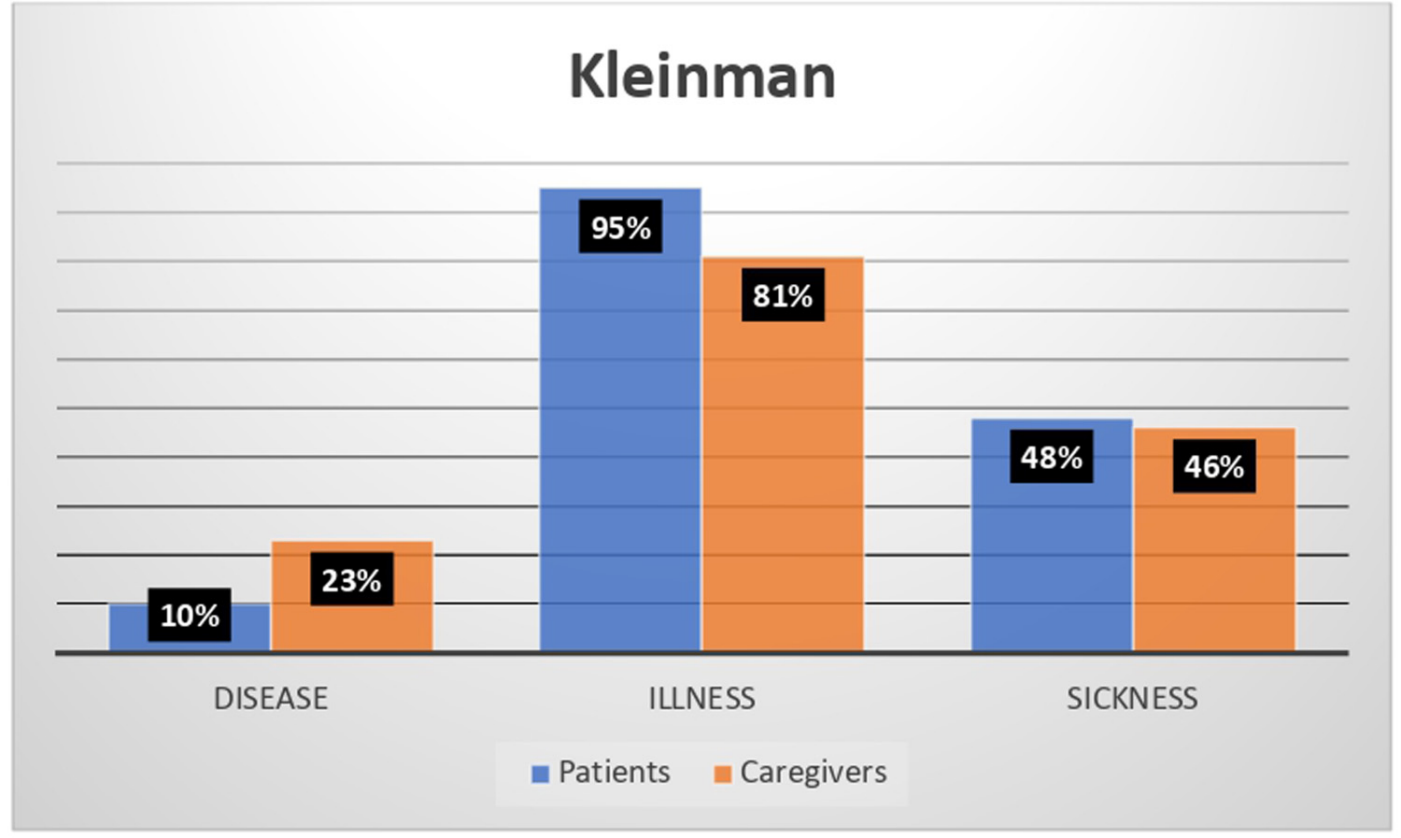
The Kleinman classification system results for how aniridia is viewed in the patient and caregiver narratives.

According to Kleinman's classification, adult patient narratives were illness-related (95%) or sickness-related (48%), more than disease-related (10%). Similarly, caregiver narratives were illness-related (81%) or sickness-related (46%), more than disease-related (23%) (Figure 1).

**Figure 2.**
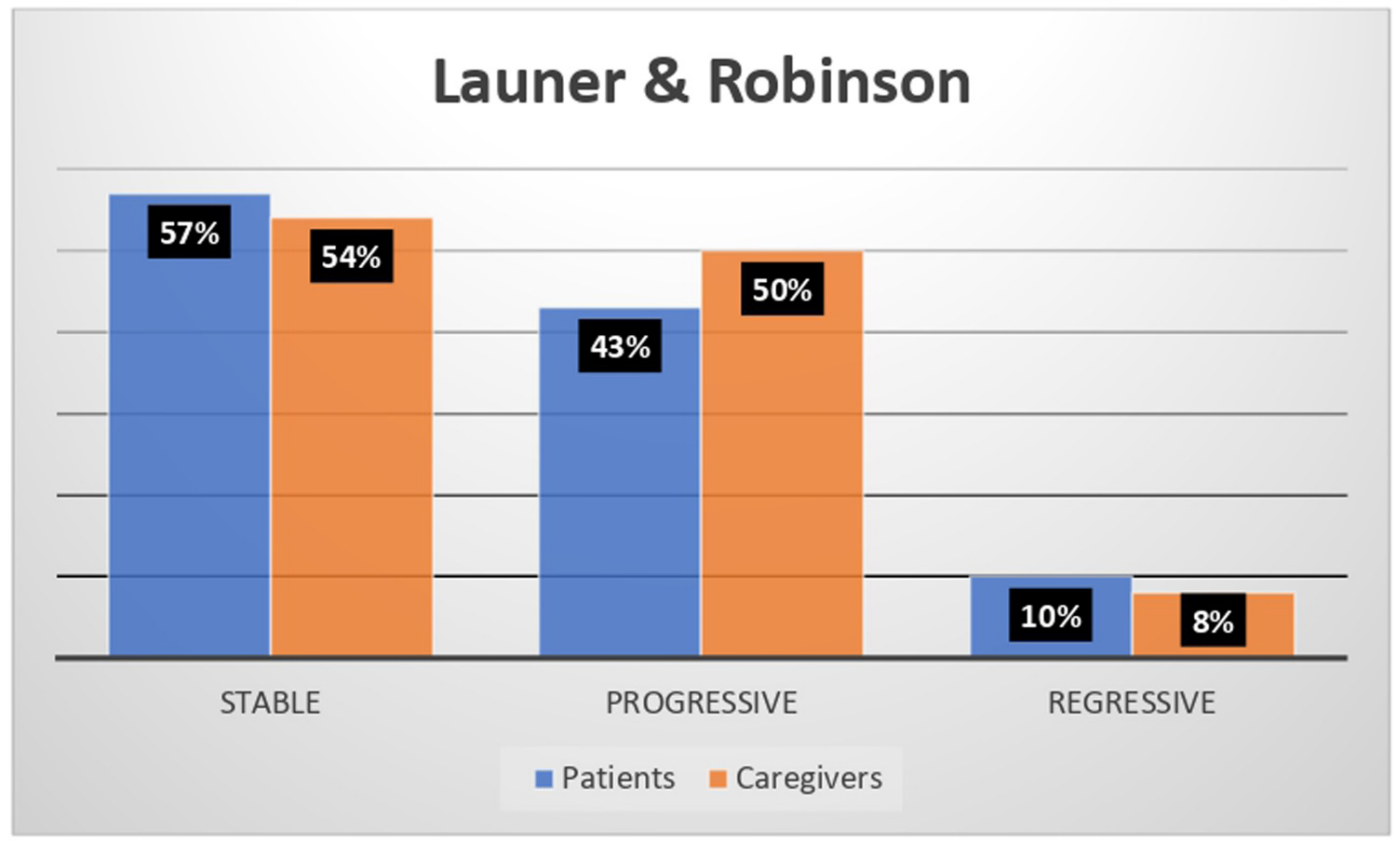
The Launer & Robinson classification system results revealing the patient and caregiver approach to aniridia over time.

The analysis of the narratives of adult patients and caregivers according to Launer and Robinson's classification revealed a stable (57% and 54% respectively) or progressive (43% and 50% respectively) rather than regressive (10% and 8% respectively) approach to aniridia over time (Figure 2).

### Macro-themes

Three macro-themes were identified transversally in both adult patient and caregiver narratives. Table 1 illustrates some examples.

1. Independence. This theme was recurrent in 43% of narratives of adult patients and in 38% narratives of caregivers. Examples are in the following quotes: “*Seeing is to be independent”* and “*I would like her to become an independent adult who accepts her condition”*.
2. Feeling misunderstood or judged. This theme recurred in 43% of adult patient narratives and in 23% of caregiver narratives. Examples are in the following quotes: “*The people around me have prejudices. I feel lonely and without friends”* and “*People around us mostly don't really understand”*.
3. Bullying This theme recurred in 14% of adult patient narratives and in 8% of caregiver narratives. Examples are in the following quotes*: “The school years were not often cheerful, bullying due to the visual impairment were the norm”* and “*He is being bullied all the time”*.

**Table 1.** Representative responses from adults with aniridia and caregivers.

	Independence
Adult patients	***-*** *Seeing is* *… Independence*. -*Seeing is an important sense especially in today's digital world. Lots of situation with the presence of a screen. Hence the feeling of loss of autonomy and stress. The prospect of losing one's sight is very anxiety-provoking.*
Caregivers	*-I would like her to become an independent adult*. -*I will ensure that I bring up my daughter in the most autonomous and serene way possible, making her aware not of what she lacks, but of what she has, accompanying her on the path of growth towards complete independence and happiness*.
	**Feeling misunderstood or judged**
Adult patients	***-*** *Also that persons who see don't understand my circumstances*. ***-*** *I felt the judgement of others.*
Caregivers	**-** *The people around us … mostly don't really understand* -*The people around us … have difficulty in understanding what we are going through*
	**Bullying**
Adult patients	*-The school years were not often cheerful where bullying due to the visual impairment were the norm*. ***-*** *I suffered years of bullying and I went into depression.*
Caregivers	*-He is being mobbed all the time*. *-I was feeling … angry because she suffered, was bullied and felt inferior*

We also identified some relevant themes within the caregivers’ narratives:

1. The lack of a center for aniridia as a reference point, lack of uniform national or international protocols and lack of sufficiently trained doctors. This theme recurred in 50% of caregiver narratives. An example is the following quote: “*…that there was a specific center for aniridia, bringing together all the different disciplines and providing professional help. Most of the doctors we meet have never had a patient with aniridia, so sometimes they ask me which medication we should choose (!) I wish there was more knowledge about aniridia in the general health system”*.
2. The need for practical and psychological support and information, especially at the time of diagnosis. This theme was recurrent in 46% of caregiver narratives. An example is the following quote: “*…that someone had explained to me what it meant to have aniridia and fewer problems in obtaining financial aid”*.
3. The lack of humanity among doctors. The theme was recurrent in 19% of caregiver narratives. An example is the following quote “*I would have liked more humanity from doctors”*.

### Comparison of themes

Despite the unsuitability of conducting a statistical comparison between the various groups of patients due to the limited sample size of patients under 18 and under 12 (*n* = 5 for each group), an attempt was made to identify cross-cutting themes meriting reflection among patients of different age groups and has been summarized in Table 2.

**Table 2.** The table illustrates the percentage of occurrences of a given theme across the various age groups.

	8–11 yo	12–17 yo	>18 yo
Activities I like to do: SPORTS	100%	100%	48%
Activities I cannot do: DRIVING	/	40%	52%
Today I feel: HAPPY/GOOD	80%	60%	29%
People around are: KIND	60%	60%	33%

By contrast, the groups of adult patients and caregivers were more comparable, given their similar sizes (21 patients over the age of 18, and 26 caregivers). Table 3 shows how the main themes are compared.

**Table 3.** The table compares the topics most frequently mentioned by adult patients and caregivers in the same prompts.

Aniridia is…	Something to live with/ challenge	Disease	Obstacle	Complex	Aesthetics/peculiarity
>18 yo	43%	38%	19%	5%	/
Caregivers	35%	12%	4%	31%	19%
**Seeing is…**	**Everything**	**Beautiful thing/gift**	**Sense**	**Independence**	**Important for life/work**
>18 yo	29%	24%	24%	19%	/
Caregivers	23%	4%	8%	8%	27%
**For the future…**	**Eyesight**	**Research**	**Personal fulfillment**		
>18 yo	48%	43%	24%		
Caregivers	46%	35%	31%		

## Discussion

This study employs a narrative medicine approach to deeply explore the illness burden associated with congenital aniridia. COREQ guidelines were taken into account to enhance the quality and the transparency of the study, particularly with regard to the design of the study (participant selection, setting and data collection) as well as the analysis and findings. A particular strength of narrative medicine compared to other qualitative approaches lies in its explicit focus on lived experience and meaning-making. While traditional thematic or grounded theory analyses identify patterns across data, narrative medicine integrates these with a structured illness–sickness-disease framework and emphasizes the personal voice of patients and caregivers.^11^ This allows for the capture of psychosocial and existential dimensions such as resilience, independence, stigma, and bullying that are often overlooked by symptom-based qualitative research. Furthermore, NM complements clinical and epidemiological measures, bridging the gap between biomedical data and the subjective experience of illness, thereby providing actionable insights for patient-centered care.

By collecting and analyzing narratives across multiple European countries, the IRIS – Aniridia Stories project provided critical insights into the daily struggles, coping mechanisms, and social challenges faced by individuals with this rare genetic condition and their caregivers. Narrative medicine illuminated dimensions of the condition that traditional metrics often overlook. This approach reveals the emotional and social complexities of aniridia, offering practical insights to guide clinical practice and policymaking.

According to Kleinman's classification, participants predominantly conceptualized aniridia as an illness or a sickness, rather than as a disease. Their narratives focused largely on lived experience and societal interaction, rather than on biomedical details, underscoring the condition's profound social and emotional implications. In line with Launer and Robinson's model, which analyses the attitude toward aniridia over time, most narratives exhibited a “stable” or “progressive” trajectory, suggesting considerable resilience and adaptability in managing the condition.

From the narratives, a number of macro-themes emerged which could be considered as potential starting points for raising awareness of the experiences of living with aniridia and the possible solutions that could be employed. One of the most prominent themes was that of independence. This highlights a fundamental challenge faced by individuals with aniridia: achieving or maintaining self-sufficiency despite significant visual impairment. Another critical theme identified was the feeling of being misunderstood or judged. This is indicative of a broader societal issue whereby rare conditions such as congenital aniridia are not adequately understood, resulting in misconceptions, social isolation and a reduction in the level of support received from peers and members of the community. Bullying emerged as a particularly concerning theme, especially during school years.

Caregivers’ accounts emphasized structural and organizational shortcomings in the healthcare system. A recurring issue was the absence of specialized centers and adequately trained professionals, especially at the time of diagnosis. On average, families consulted 5.1 ± 5.9 (range 1–40) healthcare facilities over the course of their journey. Only 28% of adult patients and caregivers had access to a dedicated aniridia center in their place of residence. In the absence of institutional guidance, families often undertook independent efforts to identify appropriate specialists, frequently relying on online resources or patient associations.

The need for practical and psychological support, especially at the time of diagnosis, was another critical issue. The initial shock of diagnosis, coupled with the uncertainty about the future, creates a profound need for information and emotional support. Finally, the perceived lack of empathy from healthcare providers may be attributed to their limited experience with aniridia and the recognition that their arsenal of tools to combat the disease is constrained. This highlights the necessity for more compassionate care.

Although statistical limitations were noted due to sample disparities, a comparison of narratives by patient age revealed notable differences. Children appeared more optimistic and socially engaged, whereas adults emphasized functional limitations, such as the inability to drive, and expressed greater emotional distress (see Table 2).

A more effective comparison can be made between adult patients and caregivers (see Table 3). Themes were compared between the two groups in three main areas: “aniridia is”, “seeing is” and “for the future”. It is interesting to note that the two groups, when given the opportunity to complete the sentences, selected substantially the same themes. Indeed, for both groups aniridia is considered as “something to live with or a challenge”, rather than an “obstacle”. Furthermore, caregivers tended to emphasize the complexity of aniridia, which extends beyond the scope of ophthalmological pathology, perhaps because they are the primary person responsible for the comprehensive management of the patients. While caregivers perceive seeing as “everything” or at least “something important for life or work”, adults with aniridia regard it as “everything” and also as “a sense” or “independence” to a similar extent. A notable finding is the perception of sight as “beautiful or a gift”, a sentiment more frequently expressed by adult patients than by caregivers, who are likely accustomed to taking this ability for granted. In “for the future”, both groups identified the preservation of vision and concern for eyesight as their primary concerns, followed by the interest, hope or desire to contribute to research and the wish for personal success.

Despite its contributions, the study has certain limitations. The inclusion of participants from diverse national healthcare contexts may introduce variability in experiences and perceptions. Furthermore, the heterogeneity of aniridia in terms of severity, comorbidities, and age of onset complicates generalizations. Additionally, the study used convenience sampling, so the cohort may not fully represent the broader population living with congenital aniridia, and both descriptive and comparative analyses should be interpreted with caution, as they reflect participants’ experiences rather than population-level estimates. Future research would benefit from longitudinal design and patient stratification to capture more nuanced differences.

In conclusion, the IRIS – Aniridia Stories project has illuminated the profound emotional, social, and relational impacts of congenital aniridia, uncovering critical dimensions often overlooked by traditional clinical metrics. Through the lens of narrative medicine, the lived experiences of patients and caregivers have been explored, highlighting cross-cutting themes such as the pursuit of independence, feelings of misunderstanding and social judgment, and the challenge of bullying. These findings emphasize the need for support systems for children with aniridia and their families, including social and emotional support to ensure their well-being at every stage of life. It also highlights the importance of education and training for professionals working with people affected by aniridia to ensure that they are well equipped to meet the complex needs associated with the condition and emphasizes the need for an integrated, multidisciplinary approach involving collaboration between different specialists to provide more comprehensive care. There is also a need for institutional guidelines available to patients and general practitioners. The narratives demonstrated the resilience and adaptability of patients and caregivers, thus providing essential insights to improve quality of life and manage the condition more effectively. What also emerges is the need for increased medical and public awareness of congenital aniridia, alongside patients’ desire for societal acceptance and personal fulfillment through the achievement of their individual goals, which are integral to leading a meaningful life. Narrative medicine presents a useful approach to gaining a deeper understanding of the daily challenges associated with aniridia and a deeper understanding of the patient and the caregiver in their entirety. Recommendations for healthcare providers include adopting narrative practices in routine care, creating tailored support groups, and advocating for specialized centers. Future larger-scale and longitudinal studies could further advance the dialogue between clinical practice, research, and the broader community, contributing to the development of targeted, innovative solutions that enhance the well-being of individuals living with this rare genetic condition.

## Supplemental Material

sj-docx-1-ejo-10.1177_11206721251407851 - Supplemental material for Exploring the human experience of congenital aniridia: A narrative medicine approach Supplemental material, sj-docx-1-ejo-10.1177_11206721251407851 for Exploring the human experience of congenital aniridia: A narrative medicine approach by Chiara Ancona, Maria Giulia Marini, Francesco Minetti, Mária Csidey, Erika Maka, Tanja Stachon, Fabian Norbert Fries, Barbara Poli, Ivana Kildsgaard, Dominique Bremond-Gignac, Nóra Szentmáry, Neil Lagali and Vito Romano in European Journal of Ophthalmology

sj-docx-2-ejo-10.1177_11206721251407851 - Supplemental material for Exploring the human experience of congenital aniridia: A narrative medicine approach Supplemental material, sj-docx-2-ejo-10.1177_11206721251407851 for Exploring the human experience of congenital aniridia: A narrative medicine approach by Chiara Ancona, Maria Giulia Marini, Francesco Minetti, Mária Csidey, Erika Maka, Tanja Stachon, Fabian Norbert Fries, Barbara Poli, Ivana Kildsgaard, Dominique Bremond-Gignac, Nóra Szentmáry, Neil Lagali and Vito Romano in European Journal of Ophthalmology

sj-docx-3-ejo-10.1177_11206721251407851 - Supplemental material for Exploring the human experience of congenital aniridia: A narrative medicine approach Supplemental material, sj-docx-3-ejo-10.1177_11206721251407851 for Exploring the human experience of congenital aniridia: A narrative medicine approach by Chiara Ancona, Maria Giulia Marini, Francesco Minetti, Mária Csidey, Erika Maka, Tanja Stachon, Fabian Norbert Fries, Barbara Poli, Ivana Kildsgaard, Dominique Bremond-Gignac, Nóra Szentmáry, Neil Lagali and Vito Romano in European Journal of Ophthalmology

sj-docx-4-ejo-10.1177_11206721251407851 - Supplemental material for Exploring the human experience of congenital aniridia: A narrative medicine approach Supplemental material, sj-docx-4-ejo-10.1177_11206721251407851 for Exploring the human experience of congenital aniridia: A narrative medicine approach by Chiara Ancona, Maria Giulia Marini, Francesco Minetti, Mária Csidey, Erika Maka, Tanja Stachon, Fabian Norbert Fries, Barbara Poli, Ivana Kildsgaard, Dominique Bremond-Gignac, Nóra Szentmáry, Neil Lagali and Vito Romano in European Journal of Ophthalmology
