# Supplementary material for: Exploring the human experience of congenital aniridia: A narrative medicine approach
Source: Eur J Ophthalmol. 2025 Dec 16;36(3):601–8. doi: 10.1177/11206721251407851 (PMC13091921; doi:10.1177/11206721251407851)
Supplement: sj-docx-1-ejo-10.1177_11206721251407851 - Supplemental material for Exploring the human experience of congenital aniridia: A narrative medicine approach [file sj-docx-1-ejo-10.1177_11206721251407851.docx]

**APPENDIX**

NARRATIVE PROMPTS

8-11

| **I introduce myself…** |
| --- |
| **Today I feel...** |
| **Aniridia is...** |
| **Seeing is…** |
| **What I like to do…** |
| **At home, at school…** |
| **The people around me...** |
| **Doctors are...** |
| **For the future...** |
| **How did you feel narrating your story?** |

12-17

| **I introduce myself…** |
| --- |
| **As a child...** |
| **When I was told I had aniridia...** |
| **The activities I liked to do and those I could not do...** |
| **Caring and treatments were...** |
| **Today I feel...** |
| **Aniridia is...** |
| **Seeing is…** |
| **The activities I like to do and those I cannot do...** |
| **The people (e.g. my family…) do these activities for me...** |
| **At home, at school/work…** |
| **The people around me...** |
| **Caring and treatment are...** |
| **For the future...** |
| **How did you feel narrating your story?** |

>18

| **I introduce myself…** |
| --- |
| **As a child...** |
| **When I was told I had aniridia...** |
| **The activities I liked to do and those I could not do...** |
| **Caring and treatments were...** |
| **Today I feel...** |
| **Aniridia is...** |
| **Seeing is…** |
| **The people (e.g. my family…) do these activities for me...** |
| **At home, at school/work…** |
| **The people around me...** |
| **Caring and treatment are...** |
| **For the future...** |
| **How did you feel narrating your story?** |

CAREGIVERS

| **The early signs of aniridia...** |
| --- |
| **I was feeling...** |
| **When I was told he/she had aniridia...** |
| **The activities she/he liked to do and those she/he could not do...** |
| **Caring and treatments were...** |
| **Today I feel...** |
| **Today she/he feels** |
| **Aniridia is...** |
| **Seeing is…** |
| **The activities she/he likes to do and those she/he cannot do…** |
| **The activities I do for the person with Aniridia...** |
| **At home, at school/work…** |
| **The people around me...** |
| **Caring and treatment are...** |
| **For the future...** |
| **Thinking back on the care pathway of the person with aniridia I care for, I would have liked that...** |
| **How did you feel narrating your story?** |
