# Supplementary material for: Exploring the human experience of congenital aniridia: A narrative medicine approach
Source: Eur J Ophthalmol. 2025 Dec 16;36(3):601–8. doi: 10.1177/11206721251407851 (PMC13091921; doi:10.1177/11206721251407851)
Supplement: sj-docx-2-ejo-10.1177_11206721251407851 - Supplemental material for Exploring the human experience of congenital aniridia: A narrative medicine approach [file sj-docx-2-ejo-10.1177_11206721251407851.docx]

|  | **Patients** | **Caregivers** |
| --- | --- | --- |
| **Gender** |  |  |
| Female | 48% | 81% |
| Male | 48% | 15% |
| **Age** | 32.52±16.68 | 43.56±7.52 |
