# Supplementary material for: Exploring the human experience of congenital aniridia: A narrative medicine approach
Source: Eur J Ophthalmol. 2025 Dec 16;36(3):601–8. doi: 10.1177/11206721251407851 (PMC13091921; doi:10.1177/11206721251407851)
Supplement: sj-docx-3-ejo-10.1177_11206721251407851 - Supplemental material for Exploring the human experience of congenital aniridia: A narrative medicine approach [file sj-docx-3-ejo-10.1177_11206721251407851.docx]

|  | **Patients** | **Caregivers** |
| --- | --- | --- |
| **Education** |  |  |
| **<8 years old** |  |  |
| Elementary school | 100% | / |
| **Age 12-18 years old** |  |  |
| Intermediate school | 80% | / |
| Other | 20% | / |
| **Adults** |  |  |
| Elementary school | / | 4% |
| Intermediate school | 5% | 4% |
| High school | 24% | 27% |
| Bachelor/Master/PhD | 52% | 50% |
| Other | 19% | 4% |
| **Employment status** |  |  |
| Employed | 71% | 62% |
| Self-Employed | 10% | 23% |
| Retired | 5% | 4% |
| Not-working | 5% | / |
| Student | / | 4% |
| Other | 5% | 4% |
