# Supplementary material for: Exploring the human experience of congenital aniridia: A narrative medicine approach
Source: Eur J Ophthalmol. 2025 Dec 16;36(3):601–8. doi: 10.1177/11206721251407851 (PMC13091921; doi:10.1177/11206721251407851)
Supplement: sj-docx-4-ejo-10.1177_11206721251407851 - Supplemental material for Exploring the human experience of congenital aniridia: A narrative medicine approach [file sj-docx-4-ejo-10.1177_11206721251407851.docx]

|  | **Caregiver’s relationship to the patient** |
| --- | --- |
| Mother | 62% |
| Father | 15% |
| Wife | 8% |
| Sister | 4% |
| Son | 4% |
| Familiar (uncle/adoptive mother) | 4% |
